# Relevance of Blood Vessel Networks in Blast-Induced Traumatic Brain Injury

**DOI:** 10.1155/2015/928236

**Published:** 2015-10-01

**Authors:** Yi Hua, Shengmao Lin, Linxia Gu

**Affiliations:** ^1^Department of Mechanical and Materials Engineering, University of Nebraska-Lincoln, Lincoln, NE 68588-0656, USA; ^2^Nebraska Center for Materials and Nanoscience, Lincoln, NE 68588-0656, USA

## Abstract

Cerebral vasculature is a complex network that circulates blood through the brain. However, the role of this networking effect in brain dynamics has seldom been inspected. This work is to study the effects of blood vessel networks on dynamic responses of the brain under blast loading. Voronoi tessellations were implemented to represent the network of blood vessels in the brain. The brain dynamics in terms of maximum principal strain (MPS), shear strain (SS), and intracranial pressure (ICP) were monitored and compared. Results show that blood vessel networks significantly affected brain responses. The increased MPS and SS were observed within the brain embedded with vessel networks, which did not exist in the case without blood vessel networks. It is interesting to observe that the alternation of the ICP response was minimal. Moreover, the vessel diameter and density also affected brain dynamics in both MPS and SS measures. This work sheds light on the role of cerebral vasculature in blast-induced traumatic brain injury.

## 1. Introduction

Blast-induced traumatic brain injury (TBI) has been considered the signature injury of the wars in Iraq and Afghanistan [1]. It is estimated that more than 200,000 veterans returning from Iraq and Afghanistan have suffered TBIs, 69% as a result of blasts [2, 3]. Numerous studies looked into the brain injury mechanisms and remedies through* in vitro* experiments [4, 5] and modeling [6–8]. The brain is generally modeled as incompressible material without considering its integration with a complex network of blood vessels. As blood vessels are three to five orders of magnitude stiffer than the brain [9], its network is likely to impact the structural responses of the brain while the blast wave transmits into the brain. However, the role of vasculature in dynamic responses of the brain under blast loading has never been reported.

A few studies [10–12] did consider the role of vasculature in the brain responses under the impact loading (i.e., linear/rotational acceleration); however, their conclusions are contradictory. Zhang et al. developed a 2D human head finite element (FE) model with several idealized branches of cerebral arteries and observed that the inclusion of arteries in the brain led to a decrease in the peak maximum principal strain (MPS), shear strain (SS), and intracranial pressure (ICP) by 46%, 57%, and 42%, respectively [10]. It is speculated that the idealized artery branches are overestimated. On the contrary, Ho and Kleiven [11] observed the minimal role of vasculature in the brain response with 2% alteration in peak MPS. Their study was based on a 3D human head FE model with image-based major branches of cerebral arteries. However, the networking between small branches of blood vessels was neglected. Along the same line, an experimental study by Parnaik et al. [12] reached the same conclusion. They constructed a 2D aluminum cylinder to represent the coronal section of the head and used silicone gel as the brain. Isolated arteries made by silicone tubes were radially inserted into the brain. The artery-induced increases in peak SS and MPS were only 4% and 6%, respectively. We speculate that the networking of cerebral vasculature leads to the significant alternation of brain dynamics.

In this work, the influence of blood vessel networks on the dynamic responses of the brain under blast loading was investigated using the FE method. A spherical head and brain were developed within a shock tube. Voronoi tessellations were implemented to represent the network of blood vessels embedded in the brain. The blast wave-head interaction was simulated. Three commonly used injury measures, MPS, SS, and ICP, were monitored at different regions of the brain to quantify the role of blood vessel networks in brain dynamics.

## 2. Finite Element Modeling

**Figure 1:**
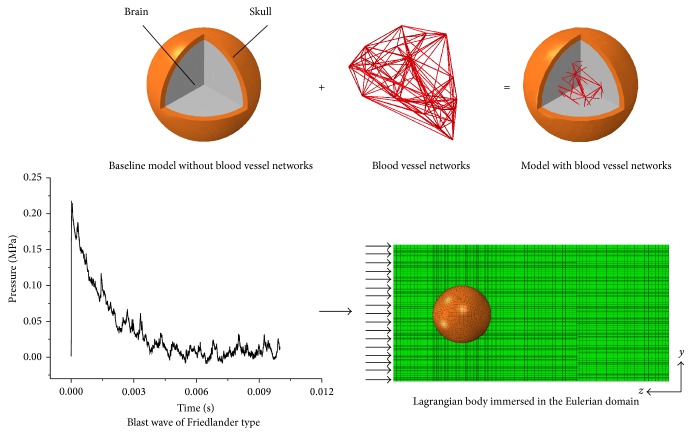
Finite element model.

The spherical head with a brain diameter of 138 mm and skull thickness of 8 mm (Figure 1) was developed to delineate the impact of the blood vessel networks [13]. Within a radius of 48 mm away from the center of the brain, 30 tessellation nodes were randomly seeded using Delaunay triangulation (MATLAB, Mathworks Corporation). Then, 161 Voronoi edges were generated to represent the vasculature network. Each blood vessel has a mean diameter of 2.72 mm, which is in the range of the reported diameter of human cerebral arteries, from 3.74 mm at the middle cerebral artery to 1.28 mm at the peripheral arteries [9]. The vasculature density, defined as the total length of the vasculature over the volume of the brain, was calculated as 0.0047 mm/mm^3^. This is comparable to the density quantification of 0.0037 mm/mm^3^ from 3D computed tomography (CT) angiographies of the human brain [11]. We used a relatively larger density to take into account the small branches of the vasculature.

The skull was modeled as a homogeneous linear elastic isotropic material with Young's modulus and Poisson's ratio of 5.37 GPa and 0.19, respectively [14]. The brain was assumed to be linear viscoelastic with a short-term shear modulus of 41 kPa and a long-term shear modulus of 7.8 kPa [15]. Young's modulus of 15 MPa and Poisson's ratio of 0.48 were adopted for the linear elastic blood vessels [10]. Air was modeled using an ideal gas equation of state since the Mach number of the blast front measured in our previous experiment [16] was approximately 1.4, and the ratio of specific heats did not change drastically at this Mach number. The material properties are summarized in Table 1.

The blast wave propagation and its interaction with the surrogate head is essentially a fluid-structure interaction (FSI) problem [17]. The air inside the shock tube was modeled with Eulerian elements, which could mimic the highly dynamic blast events. The surrogate head was modeled with Lagrangian elements. The coupling between an Eulerian domain and a Lagrangian one was enforced through a penalty contact algorithm with frictionless tangential sliding and hard contact normal behavior. The Eulerian domain consisted of 1,100,000 brick elements with approximate mesh refinement near the region of the surrogate head to capture the FSI effects. The Eulerian domain of air was chosen as 400 × 400 × 800 mm^3^ such that the reflections from the main boundaries were negligible during the 2 ms simulation time. The skull and brain were meshed with reduced eight-node hexahedral elements (C3D8R). A mesh convergence study was conducted and the mesh size of 2 mm was chosen. The blood vessels were meshed with two-node beam elements (B31) that can sustain tensile and bending loads.

The previously measured incident pressure history of a planar Friedlander waveform [16] was used as the pressure boundary condition at the inlet of the Eulerian domain (Figure 1). The velocity perpendicular to each face of the Eulerian domain was kept equal to zero to avoid escaping/leaking of air through these faces. This would create a pure 1D shock front traveling in the *z*-direction without lateral flow. The bottom of the skull was constrained in all six degrees of freedom to avoid rigid body translation. The tied constraint was used between the skull and brain. To incorporate the blood vessel networks into the surrogate head, the nodes of the vessels were merged with neighboring nodes of the brain.

## 3. Results

The computational framework has been validated against the experimental data in our previous work [16]. Briefly, repeated shock tube tests were conducted on a surrogate head, that is, a water-filled polycarbonate shell located inside the shock tube. The intracranial pressure histories at three different locations were measured. Results show that the major features of the measured pressure profiles, including the peak pressure, nonlinear decay, and small peaks and valleys, were captured by the simulation. The maximum deviation of the peak pressure in the brain was only 8.31%.

**Figure 2:**
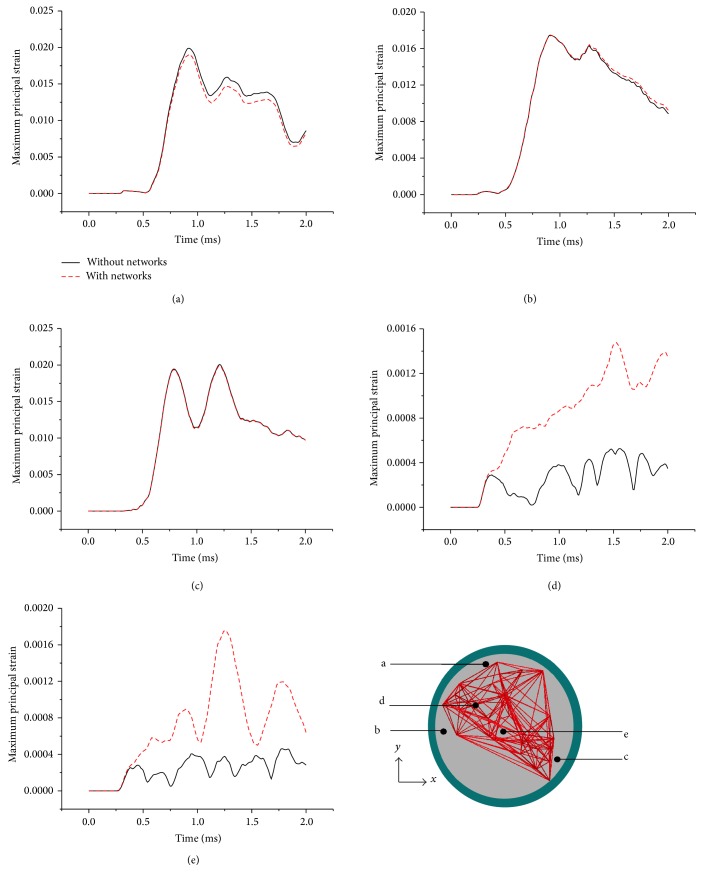
Comparison of maximum principal strain histories in five regions of the brain surrogate.

In this work, three commonly used injury measures, MPS, SS, and ICP, were monitored at different regions of the brain to quantify the role of blood vessel networks in brain dynamics. The MPS responses at five locations in the midcoronal plane of the brain were compared between two models (Figure 2). Locations represent the superior cortex (Region A), frontal cortex (Region B), occipital cortex (Region C), corpus callosum (Region D), and brainstem (Region E). The MPS magnitudes were averaged over four elements. The peak MPS predicted by the model with and without blood vessels did not differ much in Regions A, B, and C, with the maximum deviation less than 3.89%. However, in Regions D and E, the peak MPS within the model considering blood vessel networks was increased by 180.27% and 282.25%, respectively, compared to the one without blood vessel networks.

**Figure 3:**
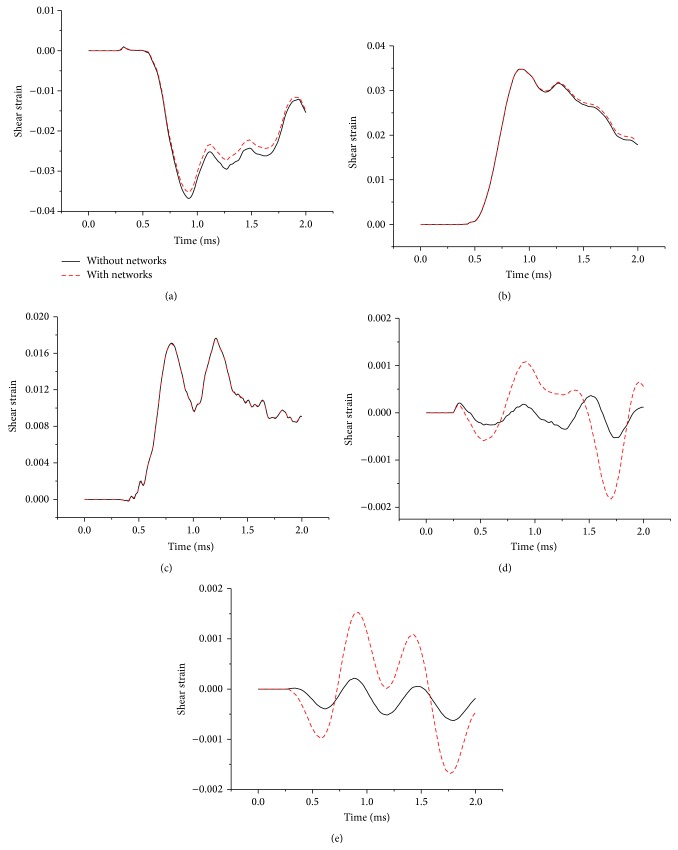
Comparison of shear strain histories in five regions of the brain surrogate.

The Green-Lagrangian SS with respect to the *y*-*z* plane is used to compare the model responses.  Figure 3 depicts the SS histories predicted by both models for the five regions described in the previous section. The predicted peak SS was as high as 3.68% and 3.53% in Region A for both models. Except for Regions A and D, all regions exhibited peak SS in the positive direction. Similar to the MPS responses, the peak SS predicted by the model with blood vessels did not differ much from the model without blood vessels in Regions A, B, and C, with the maximum deviation less than 3.94%. However, the peak SS increased by 245.28% and 612.56% in Regions D and E, respectively, for the model with blood vessel networks.

**Figure 4:**
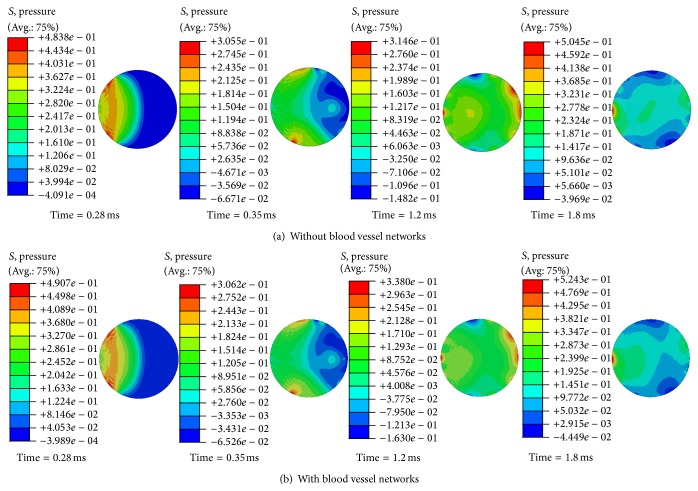
Snapshots of intracranial pressure distributions. Coup and countercoup patterns are seen at early times (time < 0.35 ms). Mixed intracranial pressure patterns are seen at later times (time > 0.35 ms).

The calculated ICP is also compared between the two models to determine the effect of modeling the blood vessel networks.  Figure 4 illustrates the ICP contours in the midcoronal plane of the brain at different times. It is observed that the inclusion of blood vessel networks did not have a significant effect on the ICP responses during the 2 ms time span. The complex wave pattern which is generated within the brain was very similar in both models. This could be attributed to the reflection of waves from finite boundaries of the head, the presence of the skull which possesses significant shear strength, and the wave mode conversion at material boundaries and interfaces. Both models exhibited typical coup and countercoup pressure patterns throughout the brain on the early time scale (time = 0.28 and 0.35 ms). Once the early waves passed through the brain, mixed ICP patterns developed at the later time scale (time > 0.35 ms). The peak coup pressures were 0.518 and 0.520 MPa and the peak countercoup pressures were −0.062 and −0.067 MPa for the models without and with blood vessels, respectively. This coup-countercoup mechanism can cause contusion at early times and can widely spread throughout the brain at later times when mixed ICP patterns are dominant.

## 4. Discussion

Two simplified head models, with and without the inclusion of blood vessel networks, were developed to quantify the effects of cerebral vasculature on the dynamic responses of the brain under blast loading. Results show that explicit modeling of the blood vessel networks could induce higher strains within the brain, specifically within the denser network region (Regions D and E) as shown in Figures 2 and 3. This is consistent with the clinical observation that the axonal bulbs were located near the blood vessels for the patients who suffered blast-induced TBI [18]. In the periphery region (Regions A, B, and C) without many blood vessel networks, the alternations in brain responses are minimal. This could be explained by the stiffening effect of blood vessel networks inside the brain. It is also interesting to observe that the ICP responses of the brain are unchanged (Figure 4), indicating that the impedance of the brain was not affected by the addition of blood vessel networks.

**Figure 5:**
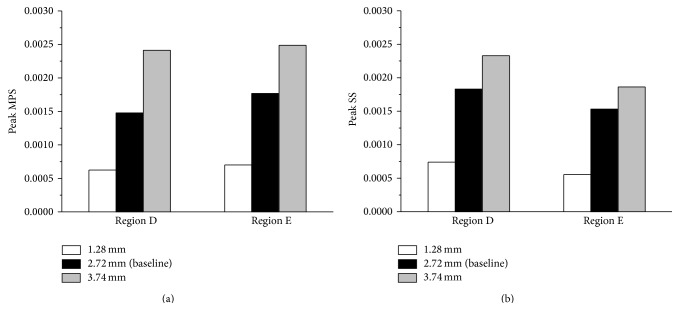
Comparison of peak maximum principal strain (MPS) and peak shear strain (SS) in the core region (Regions D and E) of the brain surrogate for different vasculature diameters.

We have assumed the blood vessel has the uniform diameter of 2.72 mm, which is approximately the averaged intracranial vessel dimension. A parametric study was performed by using two limiting intracranial vessel diameters (3.74 and 1.28 mm) measured by Monson [19] for understanding the influence of the vasculature diameter on brain dynamics. Results in terms of the peak MPS and peak SS within the embedded vessel network region (Regions D and E) of the brain are shown in Figure 5. It is clear that the peak MPS increased with the larger blood vessel size. As the blood vessel diameter increased from 1.28 to 2.72 mm, there were 57.66% and 60.38% increases in the peak MPS in Regions D and E, respectively (Figure 5(a)). When the blood vessel diameter increased from the baseline case of 2.72 mm to 3.74 mm, the peak MPS in Regions D and E increased by 63.36% and 40.83%, respectively. A similar trend is observed for the peak SS in regions D and E of the brain (Figure 5(b)). This suggests that blood vessel diameter is an influential parameter on brain dynamics. The fine vascular network with varied diameters might alter the magnitude of brain responses, but the observations on the role of blood vessel networks in brain dynamics will be the same.

**Figure 6:**
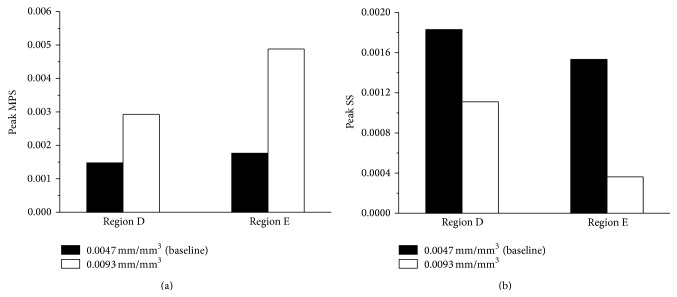
Comparison of peak maximum principal strain (MPS) and peak shear strain (SS) in the core region (Regions D and E) of the brain surrogate for different vasculature densities.

The vasculature density also varied from person to person. For example, Ho and Kleiven have quantified the intracranial vasculature density as 0.0037 mm/mm^3^ based on the CT angiographies of the human brain [11]. Parnaik et al. calculated a much higher vasculature density of 0.0093 mm/mm^3^ based on the magnetic resonance imaging [12]. We have intentionally adopted an intracranial vasculature density of 0.0047 mm/mm^3^ in our baseline model. To understand the sensitivity of brain dynamics to the vasculature density, we created a denser blood vessel network of 0.0093 mm/mm^3^ by increasing the number of tessellation nodes to 55 and the number of Voronoi edges to 342. The peak MPS and SS in Regions D and E of the brain are compared between two different vasculature densities and illustrated in Figure 6. When the vasculature density increased from 0.0047 to 0.0093 mm/mm^3^, the peak MPS in Regions D and E increased by 98.19% and 176.38%, respectively (Figure 6(a)). In contrast, there were 39.09% and 76.40% decreases in the peak SS in Regions D and E, respectively (Figure 6(b)). This opposite trend in the alternation of the peak MPS and SS might be explained by the configurations of the vascular network. Since the MPS is a predictor of diffuse axonal injuries as well as mechanical injuries to the blood-brain barrier [20, 21], this indicates that a higher density of blood vessel networks might lead to more severe brain injury. More clinical evidence could be used to examine this hypothesis.

In the present model, the human head was simplified as a spherical head. An image-based human head model incorporating layers of head materials and various brain components might lead to different stress/strain magnitudes. In addition, constitutive models for both the brain and the arteries were adopted from impact loading conditions due to a lack of testing data under higher frequency blast loading conditions, especially for the artery. Our previous work has demonstrated that brain responses dramatically decreased in terms of peak ICP, maximum shear stress, and MPS under high-frequency blast loading conditions [22]. However, considering the comparative nature of this work, the role of vessel networks in brain responses might still hold true regardless of the frequency response. Moreover, the blood vessel networks were also assumed to be Voronoi tessellations with uniform diameter located in the central region of the brain mimicking its major branches. More realistic blood vessel networks will change the peak stress and strain histories in the brain. Despite these simplifications, the present work demonstrates the importance of blood vessel networks in brain dynamics, which may have significant clinical implications for TBI.

## 5. Conclusions

The influence of blood vessel networks on the dynamic responses of the brain under blast loading was investigated using two simplified head models with and without blood vessel networks. Results have shown that blood vessel networks could influence brain responses in complex patterns. Brain dynamics are also sensitive to the dimension and density of vasculature networks. This work can be used to provide a fundamental understanding of the behavior and impact of blood vessel networks on brain responses, to provide guidance for optimizing the performance of protective equipment and to illuminate the possibilities for exploiting the potential to minimize TBI.

## Figures and Tables

**Table 1a:** (a) Elastic material properties

Material	Density (kg/m^3^)	Young's modulus (MPa)	Poisson's ratio (/)
Skull	1,710	5,370	0.19
Brain	1,040	0.123	0.499989
Blood vessels	1,040	15	0.48

**Table 1b:** (b) Viscoelastic material properties

Material	Short-term shear modulus (kPa)	Long-term shear modulus (kPa)	Decay constant (s)
Brain	41.0	7.8	0.00142857

**Table 1c:** (c) Ideal gas material parameters

Material	Density (kg/m^3^)	Gas constant (J/(kg·K))	Temperature (K)
Air	1.1607	287.05	300
